# Mouse models of intestinal cancer

**DOI:** 10.1002/path.4645

**Published:** 2015-10-29

**Authors:** Rene Jackstadt, Owen J Sansom

**Affiliations:** ^1^ Cancer Research UK Beatson InstituteGlasgowUK

**Keywords:** colorectal cancer, crc, invasion, metastasis, transplantation, GEMM, adenomatous polyposis coli, organoids

## Abstract

Murine models of intestinal cancer are powerful tools to recapitulate human intestinal cancer, understand its biology and test therapies. With recent developments identifying the importance of the tumour microenvironment and the potential for immunotherapy, autochthonous genetically engineered mouse models (GEMMs) will remain an important part of preclinical studies for the foreseeable future. This review will provide an overview of the current mouse models of intestinal cancer, from the Apc ^Min/+^ mouse, which has been used for over 25 years, to the latest ‘state‐of‐the‐art’ organoid models. We discuss here how these models have been used to define fundamental processes involved in tumour initiation and the attempts to generate metastatic models, which is the ultimate cause of cancer mortality. Together these models will provide key insights to understand this complex disease and hopefully will lead to the discovery of new therapeutic strategies. © 2015 The Authors. Pathological Society of Great Britain and Ireland.

## Introduction

In the Western world, colorectal cancer (CRC) is the second‐highest cause of cancer mortality 1. In ∼90% of fatal cases, metastasis is the cause of mortality. In the early 1990s Fearon and Vogelstein 2 postulated that mutations in CRC occur in a sequential manner, with specific mutations being associated with tumour initiation, eg the adenomatous polyposis coli (*APC*) gene, and other mutations occurring later that drive progression, eg *TP53*. Recent DNA sequencing studies confirmed the common co‐existence of these mutations within individual CRC tumours. A recent theory of CRC, referred to as the ‘Big Bang’ model, describes tumour growth as an expansion populated by various heterogeneous subclones. Initial mutations in genes, such as *APC* and *KRAS* (‘public mutations’), are carried by all subclones, and subsequent ‘private’ mutations are acquired later in individual subclones 3.

In 80–90% of CRCs the initial step is proposed to be the loss of the tumour‐suppressor gene *APC*, and this is often called the ‘classical’ route 4. Inactivation of APC induces stabilization of β‐catenin (as it can no longer be targeted for degradation) and translocation of β‐catenin to the nucleus. In the nucleus β‐catenin acts as a transcriptional co‐activator, interacting with TCF4/LEF1 transcription factors to up‐regulate expression of WNT target genes 4, 5. Another early event during tumour progression is the mutation of the proto‐oncogene *KRAS. KRAS* is mutated in 40–50% of human CRCs, with > 75% of these mutations located in codon 12, which lock *KRAS* in the active GTP‐bound state 6.

Further common mutations occur to activate the PI3 kinase signalling pathway, eg in *PTEN* or *PIK3CA*. This pathway is associated with driving cell growth, metabolism and tumour progression. TGFβ pathway abrogation in CRC can occur through mutation of either TGFβ‐receptor 1 (*TGFBR1*) or *TGFBR2*. Furthermore, TGFβ pathway inactivation can occur via loss of heterozygosity (LOH) of chromosome 18q, where *SMAD2* and *SMAD4*, two downstream mediators of TGFβ signalling, are located. Another gene deleted in colorectal cancer (*DCC*) is also localized to 18q and encodes a netrin receptor that controls differentiation and tumourigenesis 7, 8. A further late‐stage event, mainly associated with tumour cell invasion, is the mutation of the tumour‐suppressor gene *TP53* 6. Interestingly, tumours carrying *TP53* and *APC* mutations are often associated with increased rates of chromosomal instability (CIN) 9, 10, 11.

Sequencing studies have also revealed that many other mutations occur in individual CRC tumours, although at much lower frequencies (the ‘private’ mutations described above). The importance of these is still unclear and many represent passenger mutations which might have no function 12, 13, 14. Mouse models still provide the ‘gold standard’ test to see whether these mutations can functionally affect the development of cancer.

Of the remaining 20% of CRC tumours that do not carry *APC* mutations, many of these are associated with mutation of DNA mismatch repair (MMR) genes or inactivation predominantly of the mismatch repair genes *MLH1* and *MSH2* (Lynch syndrome) 15, 16, 17. These cancers have very high levels of mutation rate, evidenced by high levels of microsatellite instability, and are predominantly right‐sided and carry an improved prognosis. Recently an excellent model of Lynch syndrome has been developed through targeted deletion of Msh2 in the intestinal epithelium 18. The mutational spectra induced by a MMR defect leads to a distinct set of further mutations within these cancers. Currently it is hard to decipher the functional significance of these mutations, as they may simply be marking the DNA repair defect; however, other common mutations are found in *TGFBR2*, *ACTIVIN*, *BAX* and *MBD4*. Exciting recent data suggest that these cancers may be sensitized to immune checkpoint inhibition, potentially as a result of the higher levels of mutation 19.

Given all this information on the common mutations that occur in CRC, mouse models can be developed that are based on the genetic make‐up of tumours, generating realistic mouse models of the human disease. The successes and challenges that still need to be overcome will be the focus of this review. Due to space constraints, we have limited our review to genetic models of cancer and so do not discuss colitis‐associated cancer models within the mouse (reviewed in 20, 21). A brief overview of the models discussed in this review is provided in Table 1.

**Table 1:** Intestinal GEMMs of invasion and metastasis

**Model**	**Invasion**	**Metastasis**	**Reference**
*Apc* ^1638N/+^	Increased mucosal and submucosal invasion	Liver metastasis (1)	Fodde 42
*AhCre Apc* ^fl/+^ *Kras* ^G12V^	17% invasive carcinoma, into smooth muscle		Sansom 44
*AhCre Apc* ^fl/+^ *Pten* ^fl/fl^	32% early invasive adenocarcinomas; 22% advanced adenocarcinomas		Marsh 77
*Fabp1*Cre PIK3ca*	Invasive adenocarcinoma (analysed at day 40)		Leystra 78
*Fabp1*Cre PIK3ca* *Apc* ^Min/+^	Invasive adenocarcinoma		Deming 79
*VillinCre Apc* ^1638N/+^ *TgfbrII* ^fl/fl^	41% invasive carcinoma		Munoz 81
*Apc* ^Δ*716*/+^ Smad4^+/−^	54% submucosal invasion		Takaku 82
*Apc* ^*Min*/+^ Smad3^−/−^	Invasion to submucosa and into the muscularis propria		Sodir 83
*Apc* ^*580D*/+^ Smad2^+/−^	10–15% stroma invasion		Hamamoto 84
*Apc* ^*Min*/+^ *p53* ^−/−^	Muscularis mucosae		Halberg 87
*AhCre Apc* ^fl/+^ *p53* ^fl/fl^	25% stromal invasion		Muller 88
*AhCre Apc* ^fl/+^ *p53^R172H/+^*	100% stromal invasion		Muller 88
*Apc* ^Min/+ Δcy^ *EphB2*	100% (>30 tumours from seven different mice) classified as intramucosal adenocarcinomas		Batlle 90
*Apc* ^Min/+^ *EphB3^−/−^*	47% of the tumours were scored as invasive carcinoma		Batlle 90
*VillinCre Braf* ^LSL–V637E/+^	14% (4/29) of mice of mice showed invasive carcinoma at age 10 months	Metastasis to the mesenteric lymph nodes in 20% (1/5) of the mice	Rad 113
*VillinCre Braf* ^V637E/+^ *p53* ^R172H/+^	56% (10/18) of mice showed invasive carcinoma at age 10 months	Metastasis to the lung, pancreas, liver and mesenteric lymph nodes in 25% (3/12) of the mice	Rad 113
*VillinCre Braf* ^V637E/+^ *p16* ^−/−^	59% (20/34) of mice of mice showed invasive carcinoma at age 10 months	Metastasis to the lung, stomach, liver and local lymph nodes in 25% (3/12) of the mice	Rad 113
*VillinCre* ^ERT2^ *Apc* ^fl/+^ *Pten* ^fl/fl^ *Kras* ^G12V/+^	High‐grade invasive carcinoma in 7% of the tumours		Davies 114
*VillinCre* ^ERT2^ *Pten* ^fl/fl^ *Kras* ^G12V/+^	44% (12/27) showed invasion into the intestinal wall	41% (*n =* 11/27) present metastases; liver (7/11), pancreas (3/11), lymph nodes (2/11) and lungs (1/11)	Davies 114
*VillinCre Kras* ^G12V/+^ *TgfbrII* ^fl/fl^	70% showed marked desmoplasia and invasion	Lymph node and lung metastasis in15% (3/20)	Trobridge 115
*VillinCre Kras* ^G12V/+^ *Ink4a/Arf^−/−^*	Serrated invasive carcinoma in 76% (13/17)	Metastasis to the lung in 62% (8/13) of mice with invasive carcinoma	Bennecke 116
*VillinCre^ERT2^ Nicd1* ^LSL/+^ *p53* ^fl/fl^	59% showed invasion into muscularis and adipocyte tissue	23% (*n =* 7/30) lymph node and 10% (*n =* 3/30) liver metastases	Chanrion 121

## Mutation of APC leads to adenomas in mice

*APC* loss is the cause of familial adenomatous polyposis (FAP), a human autosomal dominant syndrome, in which patients develop numerous colorectal polyps 22, 23. Given the high prevalence of *APC* mutation in sporadic colorectal cancer and *APC* being the causal gene for FAP, most of the models developed to mimic colon cancer have centred on models carrying *APC* mutation.

**Figure 1:**
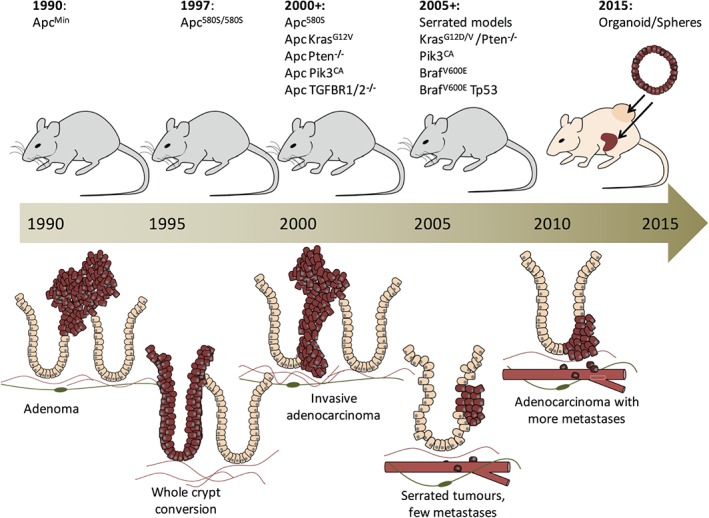
Timeline of the development of murine intestinal cancer models. The Apc^Min/+^ mouse was developed in 1990 and recapitulates the disease observed in FAP patients. In 1997, the first conditional deletion of Apc was performed in the colon and led to colonic adenomas. Acute deletion of Apc throughout the intestine led to a crypt progenitor phenotype in which whole crypts were transformed. To model more advanced disease, the Apc ^580S^ (and Apc^Min/+^) model was combined with commonly mutated oncogenes/tumour suppressor‐related genes (2000 onwards). This led to faster tumourigenesis and to increased penetrance of invasive adenocarcinomas but not metastasis. With more interest in serrated models of CRC, models driven by Kras or Braf mutations were generated. These models lacked Apc mutation and tumour latency was much increased. However, these models commonly generated adenocarcinoma that had the capacity to metastasize. Most recently, tumour‐derived and primary organoids transformed with common CRC mutations have been implanted into syngeneic or immunocompromised mice, either subcutaneously or into the kidney capsule (2015). Metastasis has been observed from tumour‐derived organoids

The most commonly used model is the multiple intestinal neoplasia (MIN) model (referred to as *Apc*^Min^ ^/+^; Figure 1) 24, 25. This autosomal dominant mutation was generated by *N*‐ethyl‐*N*‐nitrosourea (ENU) mutagenesis. The mutagen caused a loss of function mutation in the mouse *Apc* gene at codon 850. During adulthood, spontaneous LOH of the other *Apc* allele occurs and mice develop multiple intestinal adenomas and a smaller number of colonic polyps 24, 25. A major difference between the *Apc* ^Min/+^ mouse model and the human disease is that human FAP patients predominantly develop colonic lesions, whereas the mice develop more polyps in the small intestine. Furthermore, human FAP, if not treated, can progress to invasive carcinoma; this is only very rarely reported in mice, probably due to the high tumour burden in these mice and the inability to intervene surgically. The *Apc* ^Min/+^ model has been utilized for a broad range of studies; foremost have been chemoprevention studies and functional testing of genes that might modify intestinal tumourigenesis. Treatment studies of established tumours have also occurred, although it should be noted that if sporadic polyps appeared in patients, these would be removed surgically with no further treatment. Chemoprevention experiments have shown marked effects with non‐steroidal anti‐inflammatory drugs (NSAIDs), such as aspirin and celecoxib [cyclooxygenase (COX) inhibitor], although the mechanism of prevention by aspirin is likely pleiotropic and the suppression of tumourigenesis by celecoxib suggests that inhibition of COX2 is important for chemoprevention 26, 27. This work has directly translated to humans, where celecoxib reduces tumourigenesis of FAP patients and aspirin strongly reduces the risk of CRC development 28, 29, 30.

Functional genetic studies have identified numerous genes that modulate tumour development by both acceleration and deceleration. Initial studies identified modifier of MIN (*MOM)* loci through genetic linkage studies in mice. *MOM1* is located distal to chromosome 4. Interestingly, the orthologous region on the human chromosome shows frequent LOH in CRC 31. The two genes located within the mouse *MOM1* region are *Plag2g2a* and perlecan (*Hspg2*) and studies have identified that disruption of *Plag2g2a* can slow tumourigenesis 32. Further modifiers of MIN have been described and reviewed 33, 34. The identification of *MOM1* also highlighted the importance of mouse genetic background on tumourigenesis 31, 35.

*Apc* ^Min/+^ C57BL/6 J mice develop 30 polyps on average. Crossing these mice with AKR, MA or CAST strains dramatically reduces the number of polyps, indicating that the *MOM1* locus is lost in C57BL/6 J mice 35, 36. This has been tested by introducing distal chromosome 4 from AKR mice into C57BL/6 J mice 35; congenic mice showed the semi‐dominant function of the *MOM1* locus. Therefore, it is important to analyse *Apc^Min/+^* mice in a C57BL/6 J background. Otherwise, tumour burden and latency varies strongly, potentially masking the effects of the genes being tested. Many other factors can modify intestinal tumourigenesis in the *Apc^Min/+^* mouse, such as diet and the microbiome 37, 38. Recently, novel approaches have been used to discover new modifiers of tumourigenesis in the *Apc^Min/+^* mouse; sleeping beauty transposon‐mediated mutagenesis identified hundreds of alleles that can accelerate tumourigenesis in this system 14. One caveat that should be mentioned here is that if the mutation causes late‐stage progression, this might not have a phenotype in a model that only predisposes to adenoma.

Given the high penetrance of the *APC* mutation in human CRC, many other *Apc‐*truncating alleles have been generated. These include an allele, *Apc* ^1322T/+^, which very closely mimics the mutations that occur in human cancer (*APC* codon 1309) 39 and an *Apc* knockout allele that produces no protein 40. All the alleles that cause a loss of the ability of APC to bind β‐catenin lead to intestinal tumour predisposition; however, precise kinetics and tumour features can alter depending on the allele. For example, *Apc* ^1322T/+^ shows increased levels of *Lgr5* and stem cell markers within tumours, although with a slight reduction in general Wnt target gene expression, eg *Axin2* 41. Another interesting example of these mutations is *Apc* ^1638N/+^, which harbours a neomycin cassette in antisense orientation within exon 15, resulting in a protein truncated at codon 1638, which is unstable. These mice show few tumours (<10) and a long latency, and develop adenocarcinoma along with infiltration into the mucosa and submucosa 42. Thus, mice might develop tumours that more closely resemble human CRC if there were a longer latency to tumour development that allowed them to acquire further mutations that drive progression.

## Spatio‐temporal control of gene expression in vivo

The advent of *Cre–Lox* (*Cre*) technologies in the 1990s enabled researchers to delete any gene in any tissue of interest 43. In this method, mice carrying a *Cre* transgene (under the control of an inducible tissue specific promoter) are crossed to mice bearing an inducible allele where the region that is to be deleted is flanked by *LoxP* recombination sites. This can be either an essential exon(s) of a gene, to produce a conditional knockout, or a Stop motif to activate an oncogene, eg *Kras* or *Pik3*, within adult tissue 44, 45. The inducibility of Cre recombinases was most commonly achieved by coupling the Cre enzyme to the oestrogen receptor, leading to activation of *Cre* after administration of tamoxifen 46. Titration of *Cre* induction either via reducing the inducing agent (taxmoxifen/viral) or Cre recombinase also facilitates low levels of recombination, which was hoped to overcome problems of multiple tumours per mouse 47.

## APC deletion

Acute deletion of both copies of *Apc* has revealed much about the mechanism of early tumourigenesis. Shibata *et al* 48 delivered Adenovirus–Cre to the colon and showed that deletion of both copies (*LoxP* sites flanking exon 14; *Apc* ^580S/580S^; *Apc* ^fl/fl^) was sufficient to drive colon adenomas. Using a highly penetrant inducible Cre (*AhCre*, which is driven by the *Cyp1a1* promoter and is inducible by β‐naphthoflavone and *VillinCre^ERT^*) within the small intestine (and to a lesser extent the colon), we 49 and Andreu *et al* 50 showed that *Apc* loss had a dramatic impact on intestinal homeostasis. Deletion of both copies of *Apc* results in a crypt progenitor phenotype, which is characterized by increased proliferation and altered migration and differentiation. Notably, this phenotype was mediated by the Wnt target gene *Myc* 51, 52. We and others have identified a number of Wnt‐Myc targets important for this 53, 54, 55. More recently, colon‐specific deletion of *Apc* has been achieved using a *Cdx2P–CreERT2* transgenic mouse and produced a very similar phenotype to that of deletion of *Apc* in the small intestine 56. Using constitutive or inducible colon‐specific *Cre* also overcomes the problem of small intestinal tumour burden and many different colon Cres (FABPCre, A33Cre, CDX2Cre) have all been used to delete a single copy of *Apc* and generate colonic adenomas 57, 58, 59.

The discovery of *LGR5* ^+^ intestinal stem cells (ISCs) in the small and large intestine not only led to fundamental changes in concepts on ISCs and homeostasis but also allowed us to explore the impact of deleting *Apc* in the ISCs 60. LGR5 is a G‐protein coupled receptor that binds R‐spondin and thereby enhances Wnt signalling 61. LGR5 was shown to be a ‘bona fide’ ISC marker using lineage tracing. In brief, a knock‐in *Lgr5Cre^ER^* mouse was generated and interbred with the *Rosa26^LSL–LacZ^* reporter mouse. Following Cre induction, LGR5 ISCs were able to stably generate all epithelial lineages 60. Notably, using *Lgr5–CreER* to delete *Apc* within ISCs led to rapid formation of intestinal adenomas, strongly suggesting that *LGR5* ^+^ ISCs might be the cells of origin for intestinal cancer 62. Following this study, many other stem cell markers have been identified and, using a similar *Cre* knock‐in approach, ISCs have been shown to act as cells of origin for cancer when *Apc* is deleted or a constitutive‐active β‐catenin is expressed 63, 64, 65. Together these studies showed in the mouse that ISCs are highly efficient cells of origin for cancer.

However, two studies have recently demonstrated that activation of Wnt signalling in differentiated cells results in dedifferentiation and adenoma formation 66, 67. This dedifferentiation seems to require further events, eg inflammation or another oncogenic event, in addition to deregulation of Wnt signalling. Activation of *β‐catenin* ^Δex3/+^ and the inflammatory nuclear factor‐κB (NFκB) signalling pathway, in non‐ISCs (using the *Xbp1–Cre^ER^*), led to dedifferentiation and tumour development 66. The same study demonstrated that concomitant *Apc* deletion with aberrant *Kras* ^G12D/+^ expression results again in a NF‐κB‐dependent dedifferentiation. This observation is in accordance with the ‘top‐down’ model of CRC development, which is based on the observation that early dysplastic human CRC lesions predominantly locate to the luminal part and not to the base of the crypt 68. Another study investigating the potential transformation of differentiated cells targeted *Apc* deletion to terminal differentiated tuft cells, using a tuft cell marker, DCKL1. Although *Dckl1–Cre‐*mediated loss of *Apc* alone did not lead to tumour formation, when *Apc* loss was combined with dextran sodium sulphate (DSS) treatment (to induce colitis) the mice developed tumours 67. Therefore, these studies show that mouse models can inform us about the capacity of cells to act as cells of origin for cancer. The key question that remains is whether they do so in human cancer. Further cross‐comparison with human tumours and mathematical modelling is required for us to progress beyond these ‘proof‐of‐principle’ experiments.

A fundamental drawback of *Cre*‐mediated gene inactivation is that this results in the permanent deletion of a gene, and thus it is hard to assess the sustained requirement for the initiating oncogene/tumour suppressor gene. To address the continued reliance on *Apc* loss and downstream Wnt signalling, two recent studies using doxycycline‐inducible systems have shown that, if APC expression is restored (through inducible shRNA) or an inducible active β‐catenin allele is turned off, there is complete reversion to a normal intestinal epithelium. This underlines the continued dependence on Wnt signalling 31, 69. In all situations, withdrawal of doxycycline led to down‐regulation of Wnt signalling and complete tumour ablation via differentiation. This even occurred in invasive adenocarcinomas also carrying mutations in *Tp53* and *Kras* ^*G12D*/+^ 70. Therefore, GEMMs of CRC provided excellent ‘proof of concept’ that a target remains important throughout all stages of carcinogenesis.

## Generating mouse models of adenocarcinoma carrying Apc mutation

Generating mouse models of metastatic CRC has proved to be difficult. One of the key steps towards modelling metastasis is generating murine models of invasive adenocarcinoma. Cellular invasion is a complex process in which tumour cells escape from the adhesive epithelium and cross the basement membrane, invading the smooth muscle of the intestine. This is often associated with a change in cellular shape, gain of motility and loss of E‐cadherin 71. Single‐cell migration can be achieved by epithelial cells which undergo an epithelial–mesenchymal transition (EMT), resembling a developmental process 72. This process is regulated by intercellular communication of tumour cells with their microenvironment, typically mediated by cell–cell communication via chemokines or the extracellular matrix (ECM) 73. Notably, EMT has been suggested to be a dominant process during human CRC progression 74.

As mentioned above, CRC progression follows a distinct order of serial mutations 2. Since *Apc* mutations alone do not produce invasive tumours, later mutations in the adenoma–carcinoma sequence have been added to make mouse models of CRC more patient‐relevant.

With a mutation rate of ∼40% in human CRC, *KRAS* is one of the most frequently altered genes following *APC* and is also described as an early event during progression 2. Mouse models combining mutation of *Apc* with aberrant expression of mutated *Kras* ^G12V/+^ resulted in a higher number of intestinal tumours with an increased invasion of tumour cells to the surrounding stroma 44, 75. Given the high frequency of *PTEN* and *PI3KCA* mutations in human CRC 76, both *Pten* and *Pik3ca* mutant mice have been intercrossed with mice carrying *Apc* mutation. These additional mutations rapidly accelerate tumourigenesis and increase tumour progression so that the mice develop adenocarcinomas 77. When active *Pik3ca* is expressed alone within the intestine, the mice develop invasive mucinous adenocarcinoma with no intermediate benign tumour stage 78. Expression of *Kras* ^G12D/+^ or *Kras^G12V/+^* alone does not show a similar phenotype; here the mice develop both adenoma and adenocarcinoma, but at very long latencies (>500 days) 44. Thus, in mouse models, *Apc* mutation acts as an initiator, reducing latency and increasing tumour burden. This in itself is a problem, as the mice develop multiple tumours and thus may need to be euthanized due to burden before any tumours have had the opportunity to metastasize 79.

To overcome the issue of excessive tumour burden in mouse models, low‐level recombination with *Cre*‐expressing viruses targeting the colon has been performed 48, 80. Using AdCre, Hung and colleagues developed a metastatic model of CRC, based around loss of *Apc* and *Kras* ^G12D/+^ mutation. One caveat of this model is the need for surgery, which may explain the surprising lack of uptake by the research community of what appears to be an excellent model.

Loss of TGFβ signalling is a common step during CRC progression. In the mouse, *Apc* mutation in combination with inactivation of various components of TGFβ signalling (*Tgfbr2*, *Smad2*, *Smad3* or *Smad4*) generally leads to the production of invasive adenocarcinoma, although again not metastasis 81, 82, 83, 84. *Smad3* loss in the *Apc* ^Min/+^ model also altered tumour location, as more tumours arose in the distal colon 83. One of the postulated mechanisms for how loss of TGFβ drives invasion (although it is required for processes such as EMT) is that mutations in the tumour lead to a protumourigenic tumour microenvironment. For example, the increased invasion observed in *cis‐Apc* ^Δ716/+^ *Smad4* ^+/−^ mice was suggested to be mediated by recruitment of immature myeloid cells (iMCs) from the bone marrow, leading to secretion of matrix metalloproteinases (MMPs) at the invasion front of intestinal tumours 85.

The tumour‐suppressor gene *TP53* is altered in 50–60% of human CRCs. Surprisingly, deletion of *Tp53* in an outbred mouse background did not result in increased tumour progression in the *Apc* ^Min/+^ model 86. However, when analysed in a pure C57BL6/J background, *Apc* ^Min/+^ *Tp53* ^−/−^ compound mice revealed a tendency to higher tumour burden and the development of invasive tumours 87. In human CRC, gain‐of‐function mutations of *TP53* are common, particularly *TP53* ^R175H^. Expression of a single copy of the mouse version of this mutant, *Tp53* ^R172H/+^, with deletion of a single *Apc* allele, led to invasive tumour progression in all mice 88.

Collectively, it is clear that, when tested in mice, nearly all the common human mutations lead to increased tumour progression and development of adenocarcinoma, although alone these additional mutations do not provoke rapid tumourigenesis. One interesting hypothesis is that *Apc* mutation might make it harder for tumours to become metastatic in mice. This concept arose from work on two different targets of the Wnt pathway, *Ephb2/3* and *Tiam1*. EphrinB receptors (EphB) are direct Wnt target genes that control the architecture of the normal intestinal epithelium 89. Interestingly, *EPHB2*, *EPHB3* and *EPHB4* are induced during early stages but down‐regulated during CRC progression. *Ephb3* ^−/−^ in the *Apc* ^Min/+^ mice leads to conversion of 47% of tumours to adenocarcinoma, whilst ^Δcy^ *Ephb2* deletion in the *Apc* ^Min/+^ model reduces the number of tumours formed, but those tumours exhibit increased invasion 90. Loss of *Tiam1*, a pro‐adhesive RAC–guanine nucleotide exchange factor (GEF), strongly suppresses tumourigenesis in the *Apc* ^Min/+^ mice but resultant tumours are eventually invasive. Thus, it appears (at least in mice) that induction of the Wnt signalling programme favours benign tumour formation and thus additional mutations are required to drive further progression, which may in part overcome some of the pro‐adhesive consequences of *APC* loss.

## Modelling CRC metastasis with transplantation

Transplantation models are used to test pathways involved in invasion and metastasis that might be therapeutically targetable. These xenograft (human cell line) models result in desired characteristics, such as invasion and metastasis 91. However, these characteristics are dependent on the route of inoculation. Subcutaneously injected tumour cells rarely, if ever, produce any metastases, but cells injected into the caecum, tail vein, spleen, portal vein or kidney capsule can metastasize to liver, lung and bones. Dependent on the site of injection, eg tail vein, many of the barriers that cancer cells face, which stop metastasis, such as extravasation or invasion through the basement membrane, may be lacking and it is important to remember these points. Experiments are performed in immune‐compromised mice (widely used strains are *nude* or SCID mice) 92, 93, 94, 95, 96, 97, and therefore lack a number of important tumour cell–host immune system interactions. Nevertheless, studies using human CRC cell lines have demonstrated the importance of the protumourigenic microenvironment. Orthotopically injected *TGFβ*‐over‐expressing HT29 and KM12L4a CRC cells activated IL‐11 secretion from mouse cancer‐associated fibroblasts, causing increased metastasis 98. To overcome the problem of using immune‐compromised mice, allografts of mouse CRC cell lines can be used. These have been very important for modelling immunotherapy strategies. For example, the cell lines CT26 and MCA38, which were generated from mouse colorectal tumours, have been injected orthotopically to the caecum and rectal wall of Balb/c and C57BL6/J mice, respectively, and have developed liver metastasis 93.

With the discovery of *LGR5* ^+^ stem cells in the intestine, and following the isolation of these cells, Sato *et al* 99 developed *ex vivo* organoid cultures. These ‘mini‐guts’ can be grown in a three‐dimensional (3D) manner and they build tissue‐like structures 99. Outgrowth of wild‐type spheres requires the presence of Paneth cells, which provide *LGR5* ^+^ cells with niche factors 100. These cultures therefore represent an excellent opportunity to model the mutations common in colon cancer. To manipulate gene expression in these organoids, a Cre recombinase‐inducible retrovirus vector system has been developed 101. Deletion of *Apc* in these organoids results in transformation, which is characterized by a morphological change to a more rounded spheroid shape and R‐spondin‐independent growth due to hyper‐activated Wnt signalling 102. Notably, these cells can be isolated from *Villin–Cre^ER^Apc* ^fl/fl^ crypts only 2 days after tamoxifen application to the mice. Additional mutation of *Kras* ^G12D/+^ and *Tp53* ^R172H/+^ or deletion of *Pten* ^fl/fl^ confers the ability of these spheres to grow in nude mice 66, 103, 104, 105. The multi‐hit theory proposed by Fearon and Vogelstein 2 was recapitulated in mouse organoids by simultaneous deletion of *Apc*, expression of *Kras* ^G12D/+^ and deletion of *Tp53* and *Smad4* (AKPS). These spheres have an invasive phenotype similar to that of human CRC 106.

Further validation of the sequential alteration of major pathways in CRC has now also been proved in organoids from normal human crypt stem cells, by using CRISPR/CAS9 technology 107. The resulting AKPS cells show features of invasive carcinoma when subcutaneously injected into immunocompromised mice 9. Another study described that *Apc*, *Kras*, *Smad4*, *Tp53*, *PIK3CA* ^E545K^ (AKSTP) mutant cells grow when engrafted under the kidney capsule of *Nod–scid/IL2Rγ*‐null mice. However, injection of these cells into the spleen gives rise only to micrometastases in the liver, whereas cells derived from human metastatic CRC form macrometastases. This work suggested that, in addition to the major driver mutations, further alterations are required for metastatic progression and for the outgrowth of CRC metastases in the liver 108.

Organoids may therefore help us decipher the consequences of the major mutations in CRC and be very useful in high‐throughput screening for new therapies and potential therapeutic stratification. Already, much progress has occurred in the screening of tumour organoids from humans 109, providing promise for personalized/stratified therapy. It should be noted, however, that so far most of the screening has been done with organoid cultures in Matrigel**^®^**, and it will be important to see how microenvironmental changes and the culture of spheres might alter the response of these drugs; we have shown that basic properties, such as the ratio of E‐cadherin:β‐catenin, are very different in the *in vivo* setting versus cell culture 110. These new organoid models should lead to both the reduction and replacement of animal experiments. Given the need to test therapies in a 3D environment with an intact tumour stroma, there is still a very important role for autochthonous models, but hopefully experiments performed in organoids will predict *in vivo* responses better than other model systems.

## GEMMs of metastatic intestinal cancer

One of the major goals of utilizing mouse models of cancer is to recapitulate the human disease in order to produce models to test treatments. Thus, to predict response in this setting, we need models that metastasize and these models are still lacking. Thus far, most of our more successful models of metastasis are still of long latency and low penetrance. Also, most of these models do not carry mutation of *APC*. In this section we will describe these models.

In addition to the classical model of CRC progression, alternative routes to CRC have been described 6. One alternative route is the serrated route, which is characterized by hyperplastic lesions and a saw‐toothed (serrated) histology of the intestinal epithelium 111. Molecular differences between the classical and serrated route also exist. The serrated route is characterized by initial *BRAF* or *KRAS* mutations and no *APC* mutations 112. In a mouse model of serrated CRC, the expression of oncogenic *Braf* ^LSL–V637E/+^ from its endogenous promoter led to the full progression of serrated hyperplasia to adenoma and finally to metastatic carcinoma. However, latency was long and the percentage of metastasis was low with *Braf* ^LSL–V637E/+^ alone (one of five mice). A possible increase in metastasis was detected when mutant *Tp53* ^R172H/+^ (three of 12 mice) or *p16^fl/fl^* (three of 12 mice) were also mutated in addition to *Braf* mutation, but latency and penetrance were still low 113. A further model of serrated tumourigenesis that progresses to adenocarcinoma was driven by mutation of *Kras* ^G12V/+^ and *Pten* ^fl/fl^ deletion; here, 41% of mice developed metastasis, with over half developing in the liver 114. Another model that has shown metastasis is *Kras* ^G12D/+^ mice combined with deletion of *Tgfbr2*; here, CRC cells spread to local lymph nodes and the lung in 15% of the mice. This dysplastic progression was triggered by hyper‐activated EGFR signalling 115. Lung metastasis was detected in 62% of mice with concomitant *Kras* ^G12D/+^ activation and *Ink4a/Arf^−/−^* deletion; primary invasive tumours showed serrated morphology and *p16*‐dependent depression of senescence 116. It is interesting to note that all these models have in common a long latency and a lack of *Apc* mutation. However, in all models, high levels of Wnt signalling were observed in the adenocarcinoma and metastases that arose, suggesting that Wnt activation may progress these lesions from serrated lesions into ‘bona fide’ adenocarcinomas. The relevance of these serrated models has recently come to the fore, given that CRCs which have the poorest prognosis often have a ‘serrated’ signature 117.

Notch signalling is a key regulator of intestinal epithelial cell fate during normal homeostasis and contributes to tumour development 118. Genetic alterations in the Notch pathway leading to human CRC have not been reported. However, *FBXW7* is altered in 20% of human CRCs and can control Notch receptor stability 119. The function of Notch signalling in intestinal mouse models is controversial, as over‐expression of the intracellular active domain of the Notch‐receptor 1 (*Nicd1* ^LSL–GFP^) in combination with the *Apc^Min/+^* mutation generates higher numbers of adenomas which were higher‐differentiated compared to the control 120. However it has recently been shown that aberrant expression of *Nicd1* in combination with *Tp53* deletion in the mouse intestine generates adenocarcinomas that exhibit markers of EMT. Analysis of these mice revealed that 23% had lymph node infiltration and 10% showed spread of tumour cells to the liver 121. Lymph node infiltration with an EMT of the primary tumour has also been reported when *Tp53* was deleted in IEC and mice where challenged with AOM 122. It will be therefore of interest to discover whether any of these models can produce metastasis with a higher penetrance and faster latency when further oncogenic/tumour suppressor mutations are added.

## Other species

During recent years, other animal models of CRC have been developed in both rats and pig. Both, especially the pig, can recapitulate human physiology and pharmacology in a much better way than mice. In rats, two models of CRC were developed by administration of ENU, the same mutagen used for generating the *Apc^Min/+^* mouse 123, 124. The most appropriate of these is the *Apc^Pirc/+^* rat, which harbours a mutation in *Apc* which converts lysine → Stop at codon 1137 123. These rats exhibit strikingly similar pathology to human CRC, with the development of tumours with intramuscular invasion 125, 126. The porcine model of FAP was created by generating porcine ES cells carrying an *Apc^1311^* mutation 127. Germline heterozygous pigs were developed that went on to develop multiple polyps by age of 1 year (both low‐ and high‐grade dysplasia) and so act as an excellent model of FAP. Taken together, these new models open new avenues to model early‐stage human CRC, but still lack metastasis.

## Conclusion and future work

It is 25 years since the publication of reports of the *Apc^Min/+^* mouse and this model has been extensively used to characterize the mechanism, modifiers and potential therapeutic strategies for early‐stage intestinal tumourigenesis 128. Development of models that more closely mimic late‐stage disease for routine use by the community have lagged well behind, so there is not a routine GEMM for CRC that has a short latency and high penetrance. The recent excitement over new subtypes of CRC and potential stratification of patients by mutation and/or subtype makes the need for model systems more important than ever. Moreover, as immunotherapy trials become more and more the norm in cancer research, the need for immunocompetent autochthonous models to test rational combinations is vital. The advent of organoids over the past 10 years from both mouse and human normal intestine and cancer offers excellent new model systems. Transplantation of these are currently non‐orthotopic but in the future orthotopic injection may provide new models of metastatic CRC. GEMMs will remain vital to understand how the common co‐existing mutations cooperate in a natural environment. Current challenges are to assess how stroma and microbiota affect drug response, and these will need to be performed *in situ*. While we have not succeeded so far in the development of metastatic CRC models, many fundamental discoveries have been made about stem cells, homeostasis and transformation, so the community has failed very successfully! Our future aims must be to better model, understand and treat the later stages of CRC.

## Author contributions

OJS and RJ wrote the manuscript.
